# Sporadic Abdominal Wall Desmoid type Fibromatosis: treatment paradigm after thirty two years

**DOI:** 10.1186/s12893-018-0367-6

**Published:** 2018-06-07

**Authors:** S. D. Couto Netto, F. Teixeira, C. A. M. Menegozzo, H. M. Leão-Filho, A. Albertini, F. O. Ferreira, E. H. Akaishi, E. M. Utiyama

**Affiliations:** 1 Department of Surgery, Division of General Surgery and Trauma, Hospital das Clínicas – University of São Paulo, Av. Dr. Enéas de Carvalho Aguiar, 255, São Paulo, 05403-010 Brazil; 2 Departament of Radiology and Diagnostic Imaging, São Paulo Cancer Institute (ICESP), University of São Paulo, São Paulo, Brazil; 3 São Paulo Cancer Institute (ICESP), University of São Paulo, São Paulo, Brazil

**Keywords:** Abdominal wall desmoid, Desmoid tumor, Non aggressive management, Watch and wait strategy

## Abstract

**Background:**

Desmoid-type fibromatosis is a benign mesenchymal neoplastic process. It exhibits an uncertain growth pattern and high recurrence rate. Previously radical surgical resection was the mainstay of treatment, but recently more surgeons are opting for conservative management with observation (“wait and see” policy). The authors intend to evaluate different therapeutic modalities and oncological outcomes for abdominal wall desmoid tumors.

**Methods:**

We performed a retrospective study of patients who underwent surgical, hormonal or chemotherapy treatment for abdominal wall desmoid tumors between 1982 to 2014 at two institutions affiliated with the University of São Paulo, Brazil.

**Results:**

In the study period, 32 patients were included. Twenty-seven patients had surgery upfront. Of those, 89% were women with a median age of 33 years. Mean tumor size was 10 cm. Pathology confirmed free margins in 92% of resections. Tumor recurrence rate was 11%, with median relapse-free survival being 24 months. Multivariate analysis showed that positive final margins (*p* < 0.001) and positive frozen section (*p* = 0.001) were independent predictors of recurrence. For the 5 patients who underwent pharmacological therapy, median age was 33 years and median tumor diameter before treatment was 13 cm. Four patients exhibited partial response by Response Evaluation Criteria in Solid Tumors (RECIST). The single patient who did not respond to RECIST underwent radiotherapy.

**Conclusion:**

Desmoid tumor treatment has been evolving over the past decade towards a more conservative approach. Pharmacological treatment may result in tumor size regression. When surgical excision is indicated, positive margins represent an important prognostic factor for local tumor recurrence.

## Background

Desmoid-type fibromatosis is a benign mesenchymal neoplasia with monoclonal proliferation [1]. Despite the increased recurrence rate after resection, it exhibits no metastatic potential [2]. Mac Farlane described the disease in 1832 [3]. However, the designation “desmoid” was given only in 1838 in reference to the Greek word “desmos” [4].

Desmoid-type fibromatosis is rare, comprising about 3% of soft-tissue tumors. Approximately 30% of patients have tumors related to Familial Adenomatous Polyposis (FAP), including those with a mutation on the APC gene [5]. Normally, sporadic desmoid tumors oncogenesis is associated with endocrine and physiologic factors such as estrogen hormonal stimulus and pregnancy [6–8]. Trauma and previous surgery may be related to he onset of the disease in up to 25% of the cases [2, 9–11].

Management has changed dramatically over the past decade. Radical surgery was the first line of treatment, with wide resection of the tumor and adjoining tissues. With surgical resection, local recurrence rates were between 10 and 40%. In light of this, additional treatments (radiotherapy, chemotherapy, hormonal inhibitors and non-hormonal anti-inflammatories) have been used as adjuncts or even as first line therapy with more modern conservative approaches to treatement [12–18].

The aim of this study is to analyze management approaches and outcomes for patients with sporadic abdominal wall desmoid tumors treated at two large institutions affiliated with the University of São Paulo, Brazil.

## Methods

Patients who underwent treatment for histologically confirmed abdominal wall desmoid tumor between 1982 and 2015 at Hospital das Clínicas (HC - FMUSP) and at Sao Paulo State Cancer Institute (ICESP) were retrospectively identified and analyzed. They were divided into two groups, those who were managed with surgery upfront, the surgical group (SG), and those who underwent a conservative non-surgical approach, the non-surgical group (NSG).

The information collected included the following variables: age, sex, previous pregnancy, previous scar at the desmoid location, tumor diameter, surgical margins (macroscopic incomplete, microscopic incomplete and microscopic complete resection), recurrence rate, postoperative morbidity and follow up. The status of resection margins follows the Union for International Cancer Control (UICC) R classification [19]. Since desmoid tumors exhibit an infiltrative scirrhous pattern, a microscopically positive margin may be present despite wide local resection. Given this, intraoperative frozen section is routintely performed and these results are included for analysis.

For nonoperative patients, tumor volume, method and duration of treatment, and median tumor volume reduction data were collected. In this group, response to therapy was assessed and classified according to Response Evaluation Criteria in Solid Tumors (RECIST) as complete or partial response, and stable or progressive disease.

All operations were performed by surgeons from the Sarcoma Group at each institution, and multimodal treatment was managed by clinical and radiation oncologists from ICESP.

### Statistical analysis

Quantitative data were expressed using mean, median and percentage. Categorical variables were characterized by frequency distribution. Numerical variables were represented by central tendency measures (mean and median) and variability (variance and standard deviation). Univariate analysis (Chi-Square for categorical and T-student test for continuous variables) was performed to assess statistical significance. Disease-free survival was obtained using the Kaplan-Meier method and multivariate analysis assessed independent prognostic factors for local recurrence. A *p*-value less than 0.05 was considered statistically significant.

## Results

Thirty-two patients were treated for sporadic abdominal wall desmoid tumor in both institutions during the 32-year period. Twenty-seven patients underwent operative management upfront (SG), 26 of them receiving treatment prior to 2012. The nonsurgical treatment group (NSG) comprised five patients, all managed after 2012. Patients underwent diagnostic core needle biopsy prior to receiving any therapy. Pathologic diagnosis was made by an instituational specialized sarcoma pathologist.

Table 1 summarizes the demographic data from the cohort. The majority of patients in both groups were not referrals, having their initial evaluation in our instituation.

**Table 1:** Demographic characteristics of the 32 patients according to each group.

	SG (n=27)	NSG (n=5)	
Age at diagnosis (years) Range	34 years (19 – 88)	33 years (22 – 56)	
Gender			
Female	24 (89%)	5 (100%)	
Male	3 (11%)	0	
Previous surgery	7 (26%)	1 (20%)	
Previous scar	5 (18%)	1 (20%)	
Previous pregnancy	19 (70%)	3 (60%)	
One	7 (37%)	2 (66%)	
Two	5 (26%)	1 (33%)	
Three or more	7 (37%)	0	
Disease			
Primary	25 (92.5%)	5 (100%)	
Recurrence	2 (7.5%)	0	
Surgical Margins			
Frozen Section			
R0	21 (78%)	N/A	
R1	6 (22%)	N/A	
Final margin status			
R0	25 (92.5%)	N/A	
R1	2 (7.5%)	N/A	
Tumor size (cm)		*Pre treatment*	*Post treatment*
Median	10	13.2	7,6
Range	2 - 25	9.7 – 29.9	2.8 – 23.7
Recurrence	3 (11%)		
Mean follow-up (months)	82	28.8	

During surgical resection, wide margins were targeted, with ideally two to three centimeters of disease free tissue in three dimensions. Limited resection was performed when the tumor was adjacent to critical structures (organs and major neurovascular bundles) so long as no signs of invasion were present. In 15% of the cases, ribs, bladder, and gallbladder were involved in the multivisceral resection specimen.

Frozen section detected 22% of compromised surgical margins, prompting extension of the initial resection. In the SG, final pathologic status revealed a mean tumor size of 10 cm, ranging from 2 to 25 cm, and 92.5% of microscopic free margins.

**Fig. 1:**
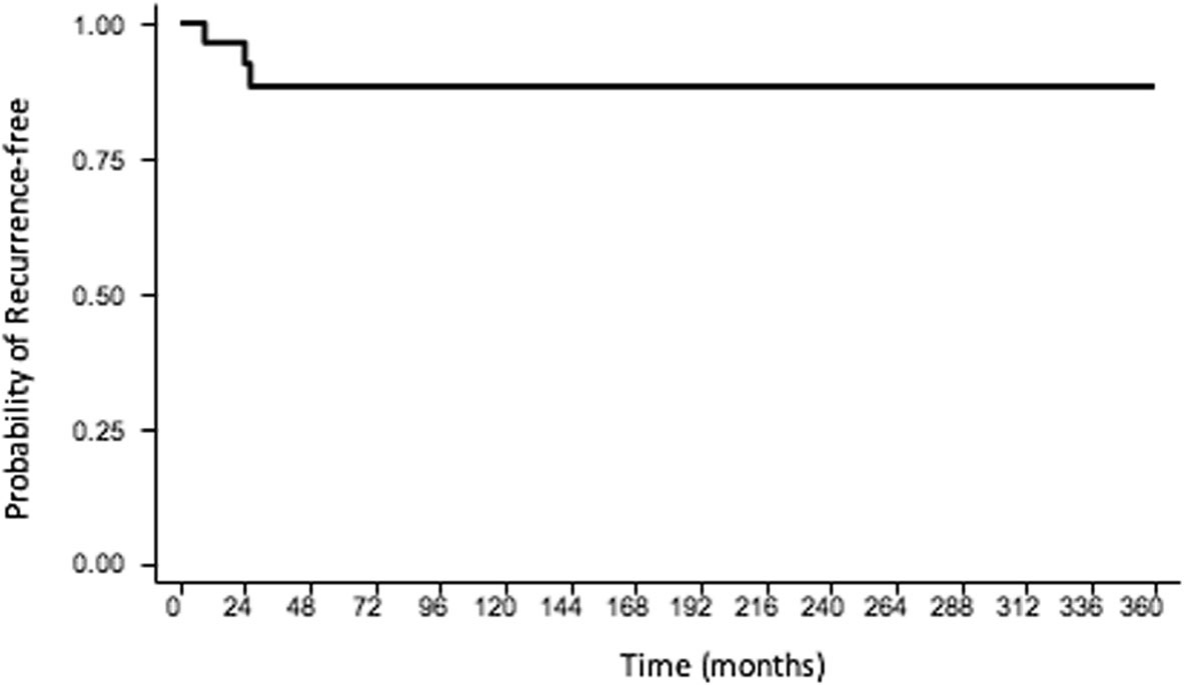
Recurrence-free overall survival of the patients who underwent surgery upfront (SG, *n* = 27)

The estimated two and five-year recurrence-free survival (RFS) rates for the entire cohort were 92 and 87% respectively (Table 2, Fig. 1). Local recurrence (LR) was observed in 3 patients (11%), including two in patients who had experience prior tumor recurrence. Mean time to recurrence was 24 months. Both patients with known compromised final margin status were already sent to our service with recurrent disease. The first patient underwent two interventions at our insituation, developed a second recurrence one year after the last procedure and doxorubicin treatment was initiated. Following this, she showed regression of the lesion size and is currently stable. The second patient exhibited recurrence 3 years after surgery. As per expert consensus following multidisciplinary tumor boards, systemic treatment was contraindicated due to the patient’s comorbidities and she was then referred to radiotherapy due to disease progression.

**Table 2:** Results of multivariable analysis of predictive factors for recurrence

	Number of patients	Recurrence-free Survival (%)		*p*-value
		2-year	5-year	
Overall recurrence-free survival	27	92.6	87.7	-
Age (years)				
< 34	14	92.6	92.6	0.514
> 34	13	100	82.6	
Diameter (cm)				
< 8	15	100	93.3	0.678
> 8	12	91.7	91.7	
Frozen section margin (n=26)				
Free	20	83.3	46.3	*0.001*
Compromised	6	100	100	
Not done	1	-	-	
Final margin status				
Free	25	0	-	*<0.001*
Compromised	2	100	95.3	
Margins upon both frozen section and final status (n=26)				
Compromised/Compromised	2	0	-	*<0.001*
Compromised/Free	4	71.4	71.4	
Free/Free	20	100	100

**Fig. 2:**
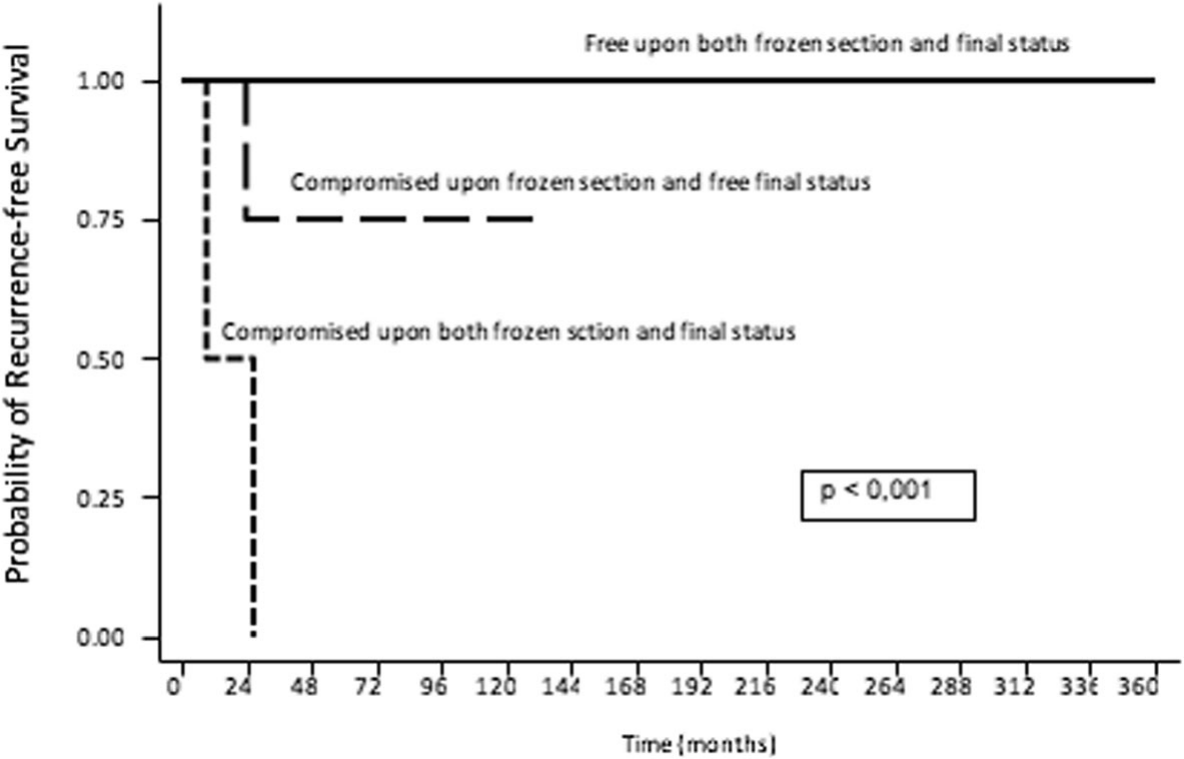
Recurrence-free survival rates according to margin status for patients who underwent surgery upfront (SG, *n* = 27)

Table 2 summarizes statistically significant findings based on multivariate analysis. Figure 2 displays the recurrence-free survival rates according to margin status. The importance of intraoperative frozen section and its results in the management of this disease is highlighted. Age and tumor diameter did not predict an increased risk of LR.

SG patients underwent abdominal wall reconstruction after tumor resection. Twenty patients had onlay polypropylene mesh placement after midline fascial closure. In the 5 patients where midline fascial closure was not feasible, a cellulose-base mesh appropriate for bowel contact was placed. Two patients had primary fascial closure with no mesh. Eight patients in SG developed some form of postoperative complication. Five had wound infection and one had deep vein thrombosis. No significant long-term morbidity was noted in these patients. There was one case of evisceration in a patient without mesh and a single case of bowel erosion in a paitent who received cellulose-based mesh.

All NSG patients in the group were from after 2012, and treatment began as a combination of nonsteroidal anti-inflammatories (NSAIDs) plus anti-estrogens (celecoxib 200 mg plus tamoxifen 40 mg) or intravenous chemotherapy (doxorubicin 75 mg/m^2^ for six cycles). The choice between regimens was based on symptoms such as pain, disease progression, and the need for a faster response rate.

Three patients were initially treated with doxorubicin 75 mg/m^2^ for six cycles, followed by tamoxifen 20 mg and celecoxib 200 mg per day was used until ideal tumor response was achieved. NSAID plus antiestrogen therapy was first line in the two other patients. Mean length of conservative treatment was 28 months (ranging from 9 to 42 months). In four patients (80%) there was a partial response, and one showed disease progression while on tamoxifen and celecoxib. This single patient was referred for radiotherapy. No patient had complete response, and mean tumor size after treatment was 7.6 cm (2.8–23.7 cm).

## Discussion

Desmoid-type fibromatosis is a clonal fibroblastic proliferative lesion which typically infiltrates surrounding structures and tends to recur, but exhibits no metastatic potential [1]. These tumors are divided into three broad groups: abdominal, extra and intraperitoneal. The latter is especially prevalent in FAP patients. Disease incidence has increased in the last few decades. While earlier reports from Reitamo et al. [20] mentioned 2 to 4 new cases per million inhabitants, a recent national database reported an incidence of 5.36 patients per million people [21].

Desmoid tumors occurs mainly in fertile young women due to hormonal influences, especially during pregnancy, or in patients with previous exposure to trauma [20, 22, 23]. As expected, in our patients group 90% were women, 62.5% had previous pregnancies, three had tumor growth during pregnancy, and 19% presented with tumors at the site of previous scars.

Based on clinical suspicion, all patients underwent core needle biopsy to establish diagnosis before initiation of any treatment. National databases have reported that desmoid tumor resection after diagnositic biopsy enhances the margin status by differentiating from other locally aggressive mesenchymal tumors [21]. Complete macroscopic resection has been the first choice of treatment for both primary tumors and recurrent disease. However, surgery alone is associated with high risk of LR ranging from less than 10% [24–28] to 40% at abdominal locations, and up to 70% in extra-abdominal disease [29]. Wide resection margins are performed with the goal of complete microscopic resection and subsequently imporved long-term outcomes, especially local recurrence.

The impact of microscopic margins on recurrence is the most controversial prognostic factor associated with desmoid tumors. Many studies from high-volume centers reveal different opinions concerning resection extension and prognosis. There is no consensus that negative microscopic margins improve local relapse [14, 30–35], however other series report the role of negative margins in predicting low or no local recurrence at all [12, 36–40]. Two desmoid tumor series from the same institution, at different times, observed conflicting results when analyzing free margins in intraoperative frozen section as independent factors associated with recurrence.

Tumor size is also another controversial prognostic factor for recurrence; several studies correlate size with local relapse. He et al. [41] analyzed 114 sporadic desmoids and observed that tumors larger than 8 cm were more likely to recur (HR: 2.43–95% CI: 1.15–5.13 *p* = 0.021). Bertani et al. [16] found that desmoid diameter greater than 10 cm predicted recurrence on univariate analysis but failed to demonstrate this with multivariate analysis (HR: 2.68 95% CI – 0.43 – 16.67 *p* = 0.29). In the largest desmoid series, Crago et al. [42], analyzed 495 patients and found that size over 10 cm was a significant independent factor for local relapse on both uni and multivariate analysis (HR: 1.94–95% CI 1.23–3.05 *p* = 0.004). However, in our study, tumors larger than 7.5 cm were not associated with local recurrence (*p* = 0.51). Correlation between size and local recurrence was not found in other studies [15, 30, 31, 38].

Age has been reported as a major factor for local relapse. In some series, lower age predicts recurrence and relapse. Crago et al. discovered more relapse in patients younger than 26 years old (HR: 4.27, *p* = 0.006) [42]. Similar findings were made by other authors such as Spear et al., who demonstrated a lower threshold of 18 years as high-risk for recurrence [32]. Sorensen et al. and He et al. observed that patients younger than 32 and 30 years-old were almost five times more likely to recur (HR: 4.97 *p* = 0.009) [33, 41]. In our series, age was not an independent factor for local recurrence, in keeping with similar results from other experienced sarcoma institutions [14, 26, 30, 34, 38, 43, 44].

Tumor location is an important prognostic factor for sporadic fibromatosis. Abdominal tumors show better prognosis than extra-abdominal desmoid tumors [15, 42, 45], and surgery alone achieves superior disease control compared to other locations in these patients [46]. Patients undergoing resection for an abdominal wall desmoid tumor have a long-term disease-free survival rate of more than 90%, whereas in young patients with large tumors located in an extremity this rate is less than 40% [29, 31, 47–52]. Large wide resections appear to be safe. Nevertheless, more extensive surgery is associated with more severe complications, early and late morbidity such as hernias, mesh complications, and the need for reoperation. Over the past ten years, there has been an ongoing trend towards intitial conservative management in large desmoid tumors or asymptomatic patients [14].

Major problems in managing desmoid tumors are their locoregional aggressiveness and their high recurrence after initial surgery, present in up to 40% of the cases [53]. Given that desmoid tumor is a benign disease and that no patient will die from it, radical surgery may be overly aggressive. In light of the considerable risk of recurrence and the potential morbidity, a conservative approach has been advocated for in the past 5 to 10 years.

**Fig. 3:**
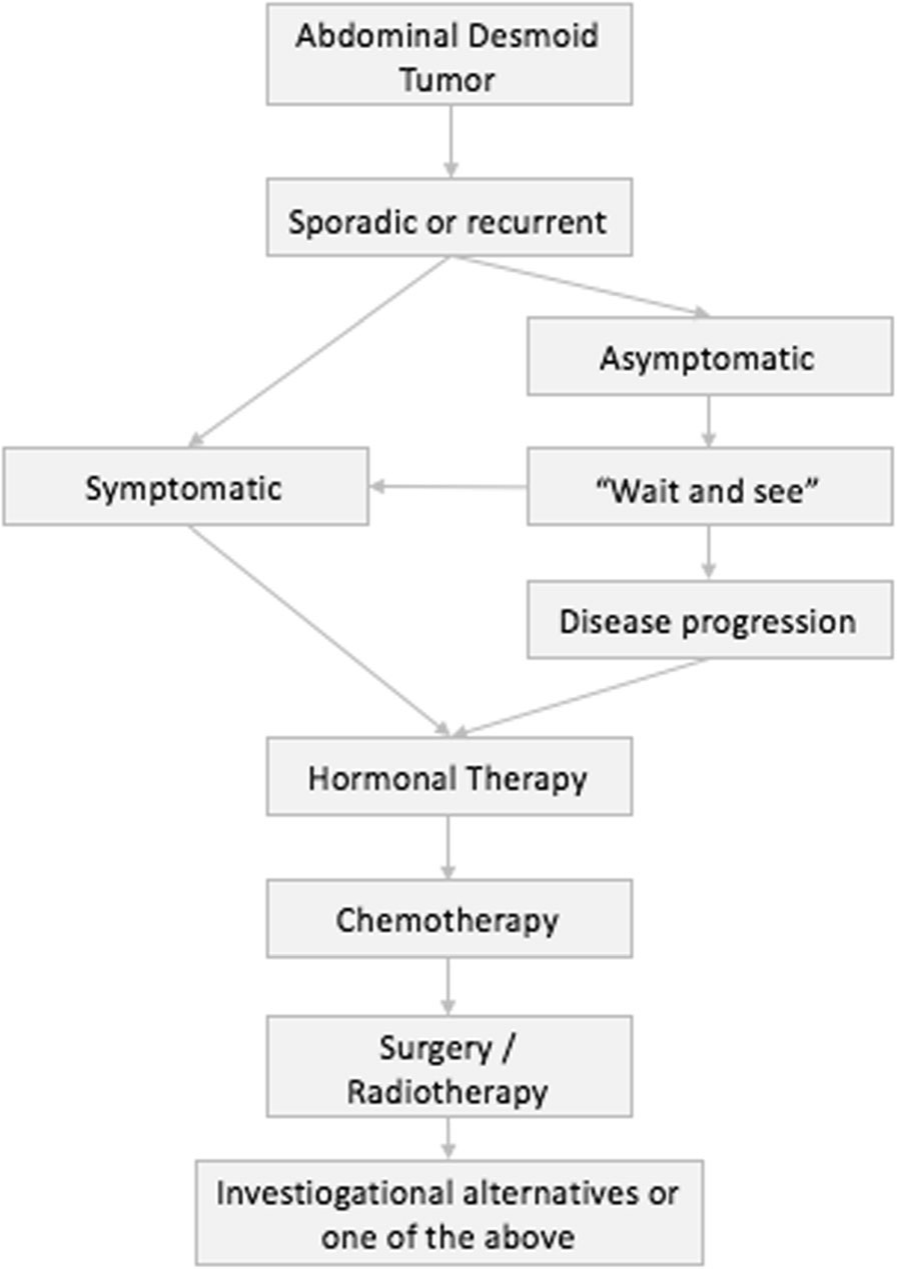
Treatment Algorithm (modified from Gronchi et al. Sporadic desmoid-type fibromatosis: A stepwise approach to a non-metastasising neoplasm - A position paper from the Italian and the French Sarcoma Group. Annals of Oncology 2014;25 [3]:578–83)

Pharmacological treatment and simple observation arose as conservative approaches. Observation is the first option in some sarcoma centers, especially after the promising results published by Bonvalot et al. in 2008 [14]. However, changes in the treatment course might occur due to symptoms of pain or disease progression. In this scenario, hormonal or even chemotherapy is the next choice of treatment. Patients with large lesions (> 7 cm) have pharmacological treatment as their first line of therapy [26]. A simple treatment algorithm already in use in some centers [54] is presented in Fig. 3.

All patients in the NSG presented with significant lesions (mean size of 13 cm), pain, and palpable masses at diagnosis. Therefore, they were all treated with chemo or hormonal therapy. Eighty percent had a partial response according to RECIST, with a mean tumor volume reduction of 31% (20 to 78%). The patient who had a major partial response by RECIST was operated on with radical intent that yielded clear microscopic margins. There was no evidence of disease after a short follow-up. In this scenario, radical surgery is not well established in the literature. There is, however, a trend offer surgery to poor responders as long as clear margins are obtainable [26].

This study is limited by its retrospective nature and its potential for selection bias, as the population submitted to surgery may be different from those allocated to the nonoperative group. However, our results show that complete microscopic removal is an independent prognostic factor for LR. Our series also reflects the historical heterogeneity of management strategies that most sarcoma centers have experienced over time.

## Conclusion

Over the past decades, there has been a change in the management of desmoid tumors towards a more conservative approach. A “wait and see” policy has been initially adopted in most cases. Medical treatment plays an important role in reducing the size of the tumors. Adoption of such treatment may avoid extensive resections and associated morbidity. When surgical resection is indicated, free margins should be pursued in order to lower the risk of local recurrence.

## Abbreviations

FAP: Familial Adenomatous Polyposis
HC-FMUSP: Hospital das Clínicas
ICESP: São Paulo State Cancer Institute
LR: Local recurrence
NSAID: nonsteroidal anti-inflammatory drugs
NSG: Non-surgery Group
RECIST: Response Evaluation Criteria In Solid Tumors
RFS: Recurrence-free survival
SG: Surgery Group
UICC: Union for International Cancer Control
